# Decoupling a tandem-repeat protein: Impact of multiple loop insertions on a modular scaffold

**DOI:** 10.1038/s41598-019-49905-4

**Published:** 2019-10-28

**Authors:** Albert Perez-Riba, Elizabeth Komives, Ewan R. G. Main, Laura S. Itzhaki

**Affiliations:** 1 Department of Pharmacology, University of Cambridge, Tennis Court Road, Cambridge, CB2 1PD UK; 2 Present Address: Donnelly Centre for Cellular & Biomolecular Research, University of Toronto, Toronto, Canada; 3 Department of Chemistry and Biochemistry, University of California, San Diego, 9500 Gilman Drive, La Jolla, CA 92093-0378 USA; 4 School of Biological and Chemical Sciences, Queen Mary University of London, Mile End Road, London, E1 4NS UK

**Keywords:** Biophysics, Molecular biophysics, Protein folding

## Abstract

The simple topology and modular architecture of tandem-repeat proteins such as tetratricopeptide repeats (TPRs) and ankyrin repeats makes them straightforward to dissect and redesign. Repeat-protein stability can be manipulated in a predictable way using site-specific mutations. Here we explore a different type of modification - loop insertion - that will enable a simple route to functionalisation of this versatile scaffold. We previously showed that a single loop insertion has a dramatically different effect on stability depending on its location in the repeat array. Here we dissect this effect by a combination of multiple and alternated loop insertions to understand the origins of the context-dependent loss in stability. We find that the scaffold is remarkably robust in that its overall structure is maintained. However, adjacent repeats are now only weakly coupled, and consequently the increase in solvent protection, and thus stability, with increasing repeat number that defines the tandem-repeat protein class is lost. Our results also provide us with a rulebook with which we can apply these principles to the design of artificial repeat proteins with precisely tuned folding landscapes and functional capabilities, thereby paving the way for their exploitation as a versatile and truly modular platform in synthetic biology.

## Introduction

Repeat proteins such as ankyrin repeats and tetratricopeptide repeats (TPRs) can be viewed as quasi one-dimensional arrays of small structural elements (typically 20–40 residues). They fold into elongated, non-globular structures that are stabilised only by local interactions whether within repeats or between adjacent repeats^1–11^. This architecture contrasts with the three-dimensional connectivity of typical globular protein, which contains many sequence-distant interactions that usually play critical roles in their folding, and it has been widely exploited in the design of repeat proteins^12–17^. The translational structural symmetry of repeat proteins is reflected in their energy landscapes^8,11^ and makes them both amenable to gross manipulation without destroying the overall structure (e.g. addition or deletion of repeats^10,14,18–20^) as well as sensitive to even small perturbations (e.g. the folding route can be redirected by single amino-acid substitutions^2,4,21–24^). The repetitive architecture also means that we can describe their thermodynamic stability using a simple one-dimensional Ising formalism. According to the Ising model, the protein is considered to be a collection of interacting units, each of which is in one of two states (folded or unfolded). Each unit is defined by an intrinsic energy term (energy difference between folded and unfolded states) and an interaction energy term (“coupling” or interfacial interaction between adjacent folded units). Thus, for artificial proteins made up of identical repeats, their thermodynamic stabilities can be adequately defined using these two parameters of the intrinsic repeat and the inter-repeat (interfacial) energies. This is referred to as a homopolymer Ising model^10,25^. The stabilities of non-identical repeat proteins can also be described using an Ising model, but a more complex description is required, which is referred to as the heteropolymer model^25–29^.

We previously used Ising models to explore the energetic consequences of extending the short (4-residue) loop between adjacent repeats of TPR proteins^29^. Strikingly, we found that the effect of loop extension is profoundly context-dependent: Extension of the central loop (between the third and fourth repeats) in a protein comprising six consensus-designed repeats (CTPR6) is much more destabilising than the same loop extension in the two-repeat protein (CTPR2). This behaviour might seem counterintuitive; however, it can be rationalised within the framework of stabilising nearest-neighbour effects of the repeat-protein architecture. Here we construct series of CTPR proteins configured with different combinations of short and long inter-repeat loops. We find that for the most extreme configuration, in which every loop of the repeat array is extended, adjacently repeats are now only very weakly coupled. Consequently, the increase in stability with repeat number, which is the defining feature of tandem-repeat proteins, breaks down. We obtain estimates of the energetics of loop extension as a function of the location and number of loops within the repeat array, and we show how dramatic this context dependence is: The cost of inserting the same 10-residue sequence ranges from as little as 0.5 kcal mol^−1^ to as much as 4 kcal mol^−1^. Our findings provide us with a “rulebook” for building artificial repeat proteins with customised energy landscapes that we can implement to functionalise this versatile scaffold, for example through the insertion of binding or catalytic loops. This truly modular toolbox thereby offers a platform for the development of designer proteins in synthetic biology.

## Results

### Design of CTPR constructs

**Figure 1:**
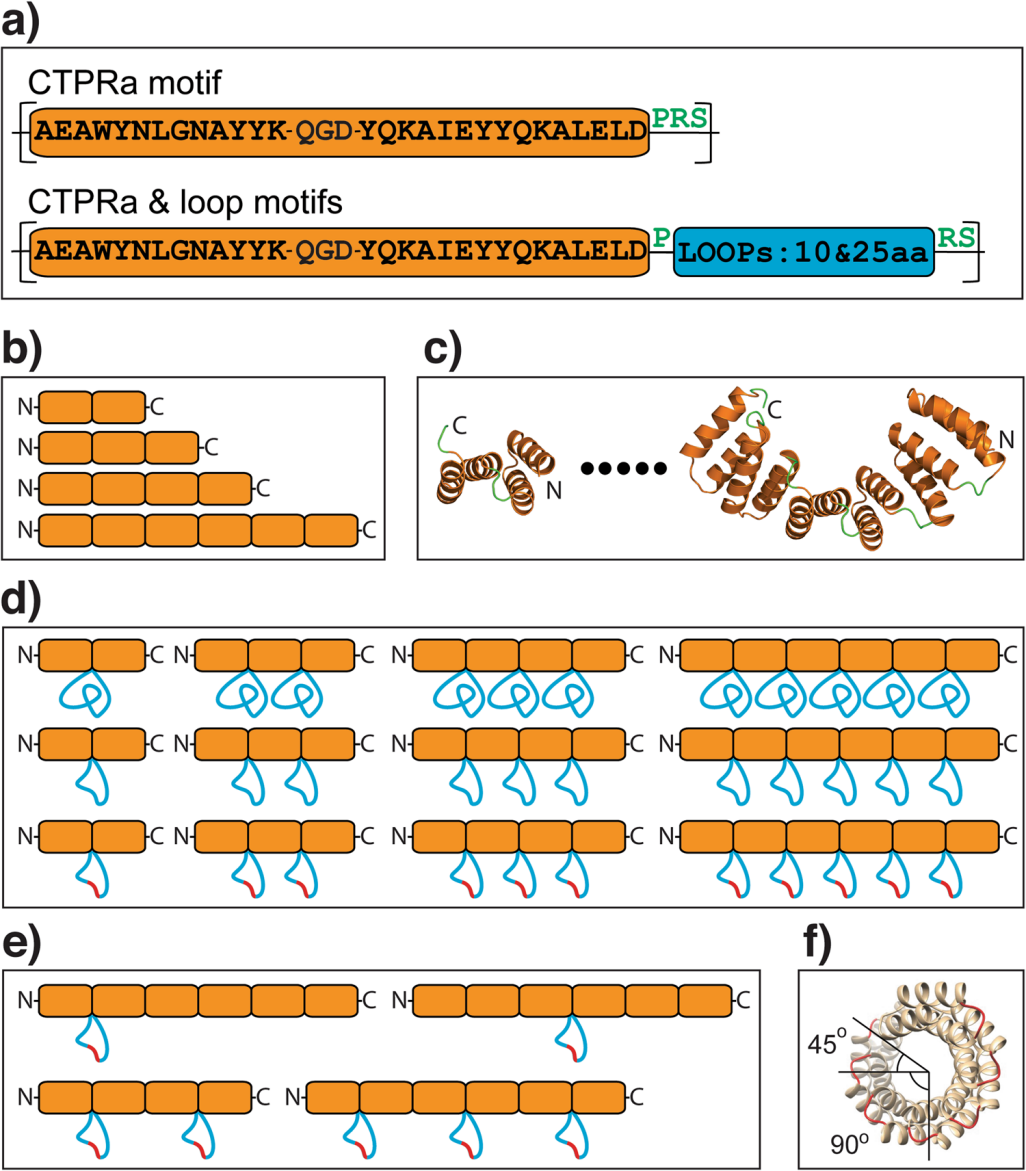
Schematics showing sequences and topologies of the proteins used in this study. (**a**) The CTPRa and CTPRa-loop motifs used. The two alpha-helices and connecting loop sequence (-QGD-) of the CTPR motif are coloured orange, with the wild-type inter repeat loop sequence (-PRS-) coloured green. The large inter-repeat loop insertions are shown as blue. Three variants of the large loops were used, one with 25-residue insertions and two with 10-residue insertions (panels D,E). (**b**) Topology of the CTPRa series of four proteins containing only the “CTPRa” motif (CTPRa2, CTPRa3, CTPRa4 and CTPRa6). Repeats are coloured as per panel (A). (**c**) Ribbon representation of the atomic structures of CTPRa2 and CTPRa6 based on the crystal structure PDB: 2HYI^31^. The dots represent the fact that this series also includes CTPRa3 & CTPRa4 (not shown). Repeats are coloured as per panel (A). (**d**) Topology of the CTPRa proteins series with multi-loop inter-repeat insertions. Each contains the same 4 proteins as the wild-type CTPRa series (panel B), with either a 25-residue loop insertion or two different 10-residue loops insertions (one has a double mutation GSLVPRGS to GSDDPRGS denoted by a red line). Repeats are coloured as per panel (A). (**e**) Topology of the CTPRa proteins series with either a single or alternate inter-repeat loop insertions. Each contains the 10-residue loop insertion with sequence: GSDDPRGS. Repeats are coloured as per panel (A). (**f**) Schematic representation of the crystal structure of an 8-repeat protein, showing that the repeats form a super-helical array and the inter-repeat loops are offset by 45° relative to each other (PDB: 2HYZ)^31^.

20 different CTPR proteins were constructed (Fig. 1). The proteins comprised two, three, four and six consensus repeats and had one of the following features: (i) wild-type inter-repeat loop sequence (DPRS) (referred to as CTPRa proteins^14,30^) (Fig. 1a-c); (ii) multi-loop variants with 10-residue or 25-residue extensions inserted into every inter-repeat loop (CTPRm proteins) (Fig. 1a,d), (iii) “alt-loop” variants, which have 10-residue extensions in alternate inter-repeat loops (CTPRalt proteins) (Fig. 1e); and (iv) a single 10-residue loop extension inserted between either the third and fourth repeats or the first and second repeats of a six repeat protein (Fig. 1e). The 10-residue and 25-residue loop extensions contained a poly(GS) sequence and a thrombin cleavage site (used previously to confirm that the loop extensions were solvent-accessible^29^). It is important to note that the simple schematics show the TPR proteins as linear but in fact they form a superhelix and therefore adjacent loops are offset relative to each other (Fig. 1f)^31^. In order to simplify our data analysis, the proteins did not contain a C-terminal capping helix (used in some previous studies^10,11,14,28–30^).

### Structure, stability and folding of 10- and 25-residue multi-loop CTPR proteins

**Figure 2:**
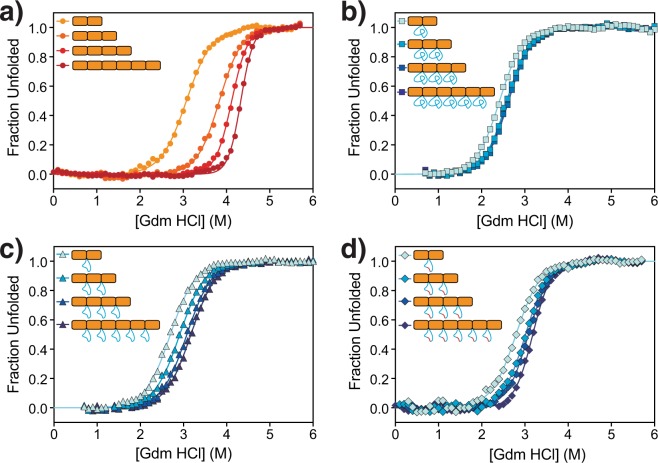
Fluorescence-monitored equilibrium denaturation curves for: (**a**) CTPRa series, (**b**) CTPRm25 series, (**c**) CTPRm10 and (**d**) CTPRm10D series. Fluorescence signal was converted to fraction unfolded for ease of comparison.

We first investigated the native structure, folding cooperativity and thermodynamic stability of the 25- and 10-residue multiloop insertion series and compared these values with those of the wild-type series (CTPRm25, CTPRm10 versus CTPRa series, respectively). Native secondary structure was characterised using far-UV circular dichroism spectroscopy (CD) (Fig. S1), with folding cooperativity and thermodynamic stability probed using chemical-induced equilibrium denaturation curves monitored by intrinsic fluorescence (Fig. 2) and CD (Fig. S2). The CD spectra indicate that all proteins had a high α-helical content, as shown by the negative ellipticity at 222 nm. Importantly, within each series of proteins the α-helical signal at 222 nm increased in rough proportion to the number of CTPR motifs in the protein (Table S2). This observation suggests that all of the proteins adopt the native TPR structure with a full complement of α-helices.

Next, the chemical-induced equilibrium denaturation curves of each series were compared (Figs 2 and S2). The results show that the CTPRa and CTPRm proteins unfold via a single transition with every multi-loop protein being destabilised relative to its wild-type CTPRa parent. To obtain a quantitative comparison, each denaturation curve was first fitted with a two-state equation to give midpoint of unfolding (D_50%_), *m*-value and free energy of unfolding (Tables 1 and S3). The data highlight a significant difference between the CTPRa proteins and the multi-loop variants. In contrast to the “normal” consensus-designed repeat CTPRa series, wherein the midpoint, *m*-value and thus stability increases with increasing repeat number, there is no such additive increase in stability for the CTPRm25 series. There is a very small increase in stability between two and three repeats (0.3 kcal mol^−1^) and no further change in stability between three, four and six repeats. The behaviour of the shorter 10-residue multi-loop series (CTPRm10) was similar to that of the CTPRm25 series (Fig. 2 and Table 1). The midpoints of unfolding increase modestly with increasing number of repeats but there is not a corresponding increase in the *m*-values, and therefore there is only a small increase in stability with increasing number of repeats. The absence of the additive increase in stability with increasing repeat number meant that neither of the multi-loop protein series could be fitted to a 1-D homopolymer or heteropolymer Ising models. In comparison, the CTPRa protein series fit well to the 1-D homopolymer model (Fig. S3), giving intrinsic and interface energies that were in agreement with previous studies (Table S4: −1.0 kcal mol^−1^ and −3.7 kcal mol^−1^, respectively)^30^. These results show that, although the introduction of multiple long inter-repeat loops into the CTPR scaffold does not cause the individual repeats to be natively unfolded, it does significantly reduce the stability and uncouples the co-operative unfolding of the CTPR proteins to an extent that is dependent on the length of the loop extensions.

**Table 1:** Parameters obtained by fitting the equilibrium denaturations monitored by fluorescence of the CTPRa, CTPRm25, CTPRm10, CTPRm10D series to a two-state folding model.

Protein Series	D_50%_ (M)	*m*-value (kcal mol^−1^ M^−1^)	${\boldsymbol{\Delta }}{{\boldsymbol{G}}}_{{\boldsymbol{D}}{\boldsymbol{-}}{\boldsymbol{N}}}^{{{\boldsymbol{H}}}_{{\bf{2}}}{\boldsymbol{O}}}$ (kcal mol^−1^)
CTPRa Series			
CTPR2a	2.97 ± 0.01	2.1 ± 0.04	6.3 ± 0.1
CTPR3a	3.76 ± 0.01	2.8 ± 0.1	10.4 ± 0.3
CTPR4a	4.04 ± 0.01	3.1 ± 0.1	12.7 ± 0.5
CTPR6a	4.35 ± 0.01	4.0 ± 0.1	17.3 ± 0.5
CTPRm25 Series			
CTPR2m25	2.41 ± 0.02	2.2 ± 0.1	5.3 ± 0.1
CTPR3m25	2.58 ± 0.01	2.2 ± 0.1	5.6 ± 0.2
CTPR4m25	2.62 ± 0.01	2.2 ± 0.1	5.7 ± 0.2
CTPR6m25	2.62 ± 0.01	2.2 ± 0.04	5.7 ± 0.1
CTPRm10 Series			
CTPR2m10	2.70 ± 0.01	2.1 ± 0.1	5.6 ± 0.1
CTPR3m10	2.90 ± 0.01	2.0 ± 0.1	5.8 ± 0.2
CTPR4m10	3.07 ± 0.02	2.0 ± 0.1	6.2 ± 0.3
CTPR6m10	3.17 ± 0.01	2.0 ± 0.1	6.4 ± 0.3
CTPRm10D Series			
CTPR2m10D	2.79 ± 0.02	2.2 ± 0.1	5.7 ± 0.3
CTPR3m10D	3.01 ± 0.02	2.3 ± 0.1	6.8 ± 0.3
CTPR4m10D	3.08 ± 0.01	2.4 ± 0.1	7.3 ± 0.2
CTPR6m10D	3.18 ± 0.01	2.8 ± 0.1	8.8 ± 0.3

Measurements for the CTPRa series were performed in triplicate, and measurements for the CTPRm25 series were performed six times. Errors in $\Delta {G}_{D-N}^{{H}_{2}0}$ were propagated from the errors obtained from the mean standard errors of the fitted variables.

### Hydrogen-deuterium exchange highlights the repeat decoupling induced by loop extension

**Figure 3:**
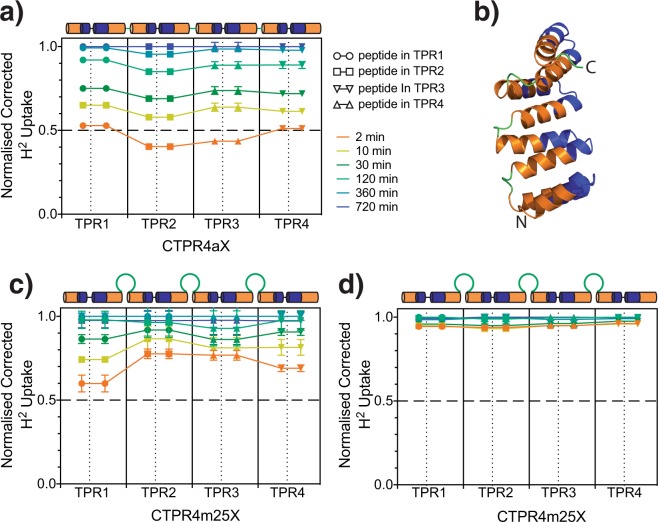
Butterfly plots of HDX MS experiments for CTPR4aX and CTPR4m25X proteins. These plots show deuterium uptake as a function of time for: (**a**) CTPR4aX, (**c**) CTPR4m25X and (**d**) denatured CTPR4m25X in 5 M urea. Each symbol corresponds to a different reporter peptide, as shown in (**b**) mapped onto a model of the CTPR4a structure as blue ribbon. Differing time points are coloured according to the legend in panel (a) and are identical in each plot. The amino-acid sequences of the reporter peptides are described in the Methods section. The x-axis of the plots represents the position of the peptide in the protein’s amino acid sequence. Above each plot, a schematic of the repeat proteins shows the secondary structure of the peptides in the protein. The deuterium uptake was corrected for back-exchange and normalised (see Methods for details of the analysis).

To explore the origin of the length-dependent uncoupling of the CTPRm proteins, we investigated the dynamics of the CTPR4m25 variant compared with its CTPR4a wild-type parent using hydrogen/deuterium exchange mass spectroscopy (HDX MS). In this approach, the protein is incubated with deuterated buffer for different times, and the samples are subsequently protease-digested into small peptides. Mass spectrometry is used subsequently to map out the solvent-protection of the amide protons and thereby gives us information on local stabilities. Our consensus-designed repeat proteins pose a challenge due to their identical repeat sequences, i.e. internal and terminal repeats will yield the same peptides upon protease digestion. Therefore, to distinguish between the repeats a small number of different point mutations were introduced into CTPR4m25 and CTPR4a (Table S5); these mutant variants are referred to as CTPR4m25X and CTPR4aX, respectively. After H/D exchange and pepsin cleavage, four of the resulting peptides were selected as representative reporters, as they covered both helices of each repeat (Fig. 3b, Table S6). The deuterium uptake for each of the peptides in the two proteins is shown in Fig. 3a,c as butterfly plots. For all peptides, the plots of deuterium uptake versus time were fitted to a double-exponential to yield two rate constants (Fig. S4). Biphasic kinetics are expected for HDX MS data, as the kinetics reflect the average behaviour of the peptide fragment rather than the behaviour of individual amino acids. The faster phase corresponds to the rapid exchange of residues that are solvent-exposed in the native proteins. The data show that the repeats of CTPR4m25X all undergo faster exchange than the repeats of CTPR4a, reflecting the lower thermodynamic stability of the former protein. Second, the internal (second and third) repeats of CTPR4aX are more protected than the terminal repeats. This result is in agreement with previous HDX NMR studies on CTPR proteins and consensus-designed ankyrin-repeat proteins, which show that internal repeats have a greater degree of solvent protection than the outer repeats^32,33^. Such behaviour reflects the hierarchical nature of repeat stability, whereby internal repeats are more stable than outer ones. In contrast, there is no such increase in protection for the multi-loop CTPR4m25X protein. The exchange rates of all repeats of CTPR4m25X are the same within error, and they can be fitted globally using shared rate constants for each of the two phases (Fig S5). The absence of enhanced protection of internal relative to terminal repeats shows that the insertion of loops causes a more dynamic native structure that undergoes more substantial local “breathing”. This conclusion is further supported by an increase in the relative amplitude of the fast phase for CTPR4m25X compared with CTPR4aX. Importantly, however, when chemically denatured CTPR4m25X undergoes H/D exchange there is very low protection in comparison to native CTPR4m25X (Figs 3d and S5). Thus, even though the inter-repeat interfaces of CTPR4m25 are more dynamic than wild-type, the global tertiary structure is retained. This conclusion is consistent with the fluorescence- and CD-monitored denaturation results.

### Sequence-dependent effects of loop extension

There are two possible, mutually non-exclusive explanations for the increased dynamics/local unfolding of the internal repeats in the multi-loop CTPR proteins relative to the CTPRa proteins. One is the entropic cost of loop insertion on the inter-repeat interface. The other is that there are steric clashes between the loops and/or between the loops and the helical TPR core. Both would prevent the characteristic build-up in stability with increasing repeat number and would lead to a loss of cooperative folding. To investigate these possibilities, we next constructed two further sets of multi-loop CTPR proteins, one with a modified loop sequence and the other with alternating short (consensus) loops and extended loops.

We reasoned that, if the effects observed are due to steric clashes, they might be sequence dependent. We noted also that the CTPRm proteins showed a decrease in solubility with increasing number of repeats. Therefore, two hydrophobic residues within the 10-residue loop were substituted for aspartate residues (GSLVPRGS to GSDDPRGS) both to test the hypothesis and to aid solubility.

This new 10-residue multi-loop series (CTPRm10D comprising 2-, 3-, 4- and 6-repeat proteins) showed greatly improved solubility compared with the original CTPRm10 series. For example, CTPR6m10D expressed in the soluble fraction with yields of over 130 mg/L of culture (compared with negligible amounts of CTPR6m10 in the soluble fraction). All proteins were found to be folded (as shown by their far-UV CD spectra; Fig. S1c), and the equilibrium chemical denaturation curves monitored by fluorescence and CD were very similar to those of the respective parent (CTPRm10) proteins (Fig. 2, Table 1, Fig. S2 and Table S3). Thus, energetic cost of multi-loop extension does not appear to be sequence-dependent and is more likely to be an entropic effect.

### Context dependence of loop extensions

As a further test of this model, we made a further multi-loop series in which alternate loops were extended to see the effect on the build-up of global stability with repeat number. This series should tell us whether the breakdown in the Ising-like behaviour of the CTPRm proteins is due solely to the entropic penalty of closing the long loop in order to form the inter-repeat interface or whether there is an additional effect of steric clashes between the loops. CTPR proteins with extensions in alternate inter-repeat loops were constructed. We used the CTPRm10D sequence, as this sequence had improved solubility relative to the original loop CTPRm10 sequence. Taking the CTPR2m10D as a “module” to be repeated twice and thrice, we created a CTPR4 protein with two extended loops (CTPR4alt10D) and a CTPR6 protein with three extended loops (CTPR6alt10D), respectively (Fig. 1e).

**Figure 4:**
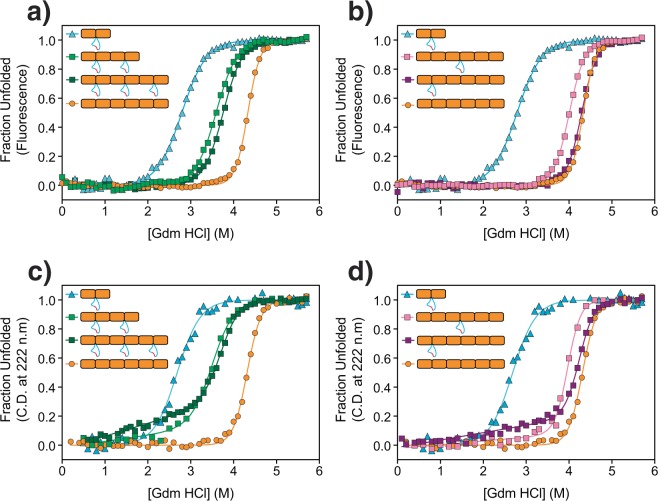
Denaturation curves monitored by fluorescence and CD for CTPRalt10D, single-loop CTPR and “wild-type” parent CTPRa proteins. (**a**) Normalised fluorescence of CTPR2m10D, CTPR4alt10D, CTPR6alt10D & CTPR6a, (**b**) Normalised fluorescence of CTPR2m10D, CTPR6L1-2, CTPR6L3-4 & CTPR6a, (**c**) Normalised ellipticity at 222 nm of CTPR2m10D, CTPR4alt10D, CTPR6alt10D & CTPR6a and (**d**) Normalised ellipticity at 222 nm of CTPR2m10D, CTPR6L1-2, CTPR6L3-4 & CTPR6a. The plots are fitted to a two-state model to guide the eye.

Fluorescence-monitored equilibrium denaturation experiments show that there is a significant increase in stability of the four-repeat array comprising two CTPR2m10D modules relative to the two-repeat array comprising only one module (Fig. 4a and Table S7). This behaviour is as expected given that CTPR4alt10D contains a native non-loop extended CTPR interface between the second and third repeats. Addition of a third module (i.e. CTPR6 with three extended loops) shows only a marginal further increase in stability. The CD-monitored denaturation curves reveal that there is a significant loss in ellipticity of CTPR4alt10D and CTPR6alt10D proteins before the major unfolding transition (Fig. 4c). This behaviour is consistent with the loop-extended inter-repeat interfaces unfolding at lower denaturant concentrations than the consensus interfaces. The midpoint of the major unfolding transition is same for fluorescence- and CD-monitored curves (Table S7).

Last, we compared a six-repeat protein having a single loop extension located between the first and second repeats (CTPR6L1-2) and compared this protein with a six-repeat protein having a single loop extension located between the third and fourth repeats (CTPR6L3-4, characterised previously in^29^). The Ising model of build-up of stability with increasing number of repeats would predict that the internal loop extension would have the larger energetic cost. In Fig. 4 we compare the equilibrium denaturation curves of these two single-loop 6-repeat proteins with those of CTPR6a and CTPR2a (Fig. 4b,d) and the two alt-loop proteins (CTPR4alt10D and CTPR6alt10D) (Fig. 4a,c), monitored both by fluorescence (Fig. 4 top) and CD (Fig. 4 bottom). The fluorescence data indicate that CTPR6L3-4 is significantly destabilised relative to CTPR6a (~3.5 kcal mol^−1^ from a two-state fit), whereas CTPR6L1-2 appears to have the same stability as CTPR6a within error (Table S7). However, the CD data reveal a more complex scenario for CTPR6L1-2: There is a loss of helical structure at low denaturant concentration, which is consistent with the unfolding of the first repeat resulting in an intermediate comprising five folded repeats. This partial unfolding is similar to what is seen for the alt-loop proteins and is consistent with the loop-extended inter-repeat interfaces unfolding at lower denaturant concentrations than the consensus interfaces.

Thus, although every loop in every protein in Fig. 4 has the same sequence and is located between repeats of identical sequence, the energetic cost of loop extension varies dramatically and is dependent on the “local stability” associated with its location in the array. We have used the two-state fits of the fluorescence-monitored denaturation curves to compile estimates of these context-specific energy penalties (Table 2). We find that the cost per loop extension is as little as ~0.5 kcal mol^−1^ in a two-repeat array, *versus* 2–2.4 kcal mol^−1^ for alternate loops (whether in four-repeat or six-repeat array), and 4 kcal mol^−1^ for the innermost loop in a six-repeat array.

**Table 2:** Energetic cost of loop insertion is dependent on location and context within the repeat array.

Protein and loop location and context		Estimated energetic cost per loop insertion (kcal mol^−1^)
CTPR2		0.6
CTPR6, L3-4		4.2
CTPR4, alt loop		2.0
CTPR6, alt loop		2.4

For each protein, the energetic cost per loop insertion was estimated by subtraction of the free energy of unfolding of the protein from that of the respective loop-free CTPR protein and then dividing by the total number of loops in the protein. Thus: CTPR2 = CTPR2a-CTPR2m10D.

CTPR6, L3-4 = CTPR6a- CTPR6L3-4.

CTRP4, alt loop = (CTPR4a-CTPR4alt10D)/2.

CTPR6, alt loop = (CTPR6a-CTPR6alt10D)/3.

Free energies of unfolding are taken from Tables S3 and S7.

## Discussion

Here we investigated the relationship between the changes in global stability, folding cooperativity and local dynamics that result from the introduction of long loops into repeat-protein arrays. The cooperativity of repeat protein folding is determined by the difference between the intrinsic folding free energies of the repeats (Δ*G*_i_) and the interfacial free energies (Δ*G*_ij_). Thus, proteins with intrinsically unstable repeats and very stable interfaces tend to unfold with high cooperativity^25^. This is typically the situation for small naturally occurring repeat proteins and their consensus-derived counterparts. Nature may have selected for proteins with high cooperativity to avoid the population of partly folded intermediates that can lead to misfolding or aggregation. In contrast, Geiger-Schuller *et. al*. recently determined the Ising parameters of a collection of *de novo* Rosetta-designed repeat proteins^34^. They found that all of these proteins had Δ*G*_i_ of −1.4 to −3.5 kcal mol^−1^ and Δ*G*_ij_ of −4.8 to −10 kcal mol^−1^. Thus, in contrast to Nature, Rosetta has optimised both the intrinsic and the interfacial free energies. The question of the balance between these two parameters is key to our study: A repeat array with low cooperativity will respond to interface disruption differently from a repeat array with high cooperativity. The consensus-designed TPR sequence used here has Δ*G*_i_ of −1 kcal mol^−1^ and Δ*G*_ij_ of −3.7 kcal mol^−1^, and these proteins are consequently less cooperatively folded than consensus-designed ankyrin repeats (Δ*G*_i_ of +4.4 kcal mol^−1^ and Δ*G*_ij_ of −11.2 kcal mol^−1^) and CTPR sequences used elsewhere (Δ*G*_i_ of +1.4 kcal mol^−1^ and Δ*G*_ij_ of −4.3 kcal mol^−1^). Loop insertion makes our rather low-cooperativity system even less cooperative but does not compromise the global native structure because the intrinsic repeat free energy, at −1 kcal mol^−1^, is mildly stabilising and only a single interface (i.e. two repeats) is required for an independent folding unit. However, for proteins with intrinsically very unstable repeats that require more than two inter-repeat interfaces to form a stable unit, loop extension might prevent folding altogether.

Loop insertion reduces the interfacial free energy because of the entropic penalty of closing that loop^35–37^. In the multi-loop CTPR series, the result is natively structured arrays that show little to no increase in global stability with increasing number of repeats (and these series are, consequently, not amenable to Ising analysis). Consistent with this behaviour, the HDX MS results show that the internal repeats are not more protected than the terminal repeats, in striking contrast to what is expected for repeat proteins^7,32,33,38,39^.

In conclusion: (1) Proteins containing long sequence insertions at every inter-CTPR inter-repeat loop are folded and stable, but the multiple insertions reduce the cooperativity and thereby the build-up in thermodynamic stabilities with increasing number of repeats that characterise the repeat-protein class; (2) The energetic cost of loop insertion is highly context dependent because the local stability of each repeat within an array is also context dependent; consequently, the cost of an inserted loop determined for one CTPR array cannot be generalised to any repeat in any size of CTPR array but rather is dependent on the length of the array and the location of the insertion along the array. Importantly, the stability costs are unlikely to be prohibitive for future applications of loop-extended CTPR proteins because the CTPR scaffold start from such high global stability to begin with. For example, the most extreme case in our study, the multi-loop extended six-repeat protein CTPR6m10D is still a highly soluble and stable protein with a melting temperature of over 80 °C (Fig. S6) even though almost half of its polypeptide chain consists of inter-repeat loops. CTPR6ml10D is, therefore, more than adequate as a scaffold for biotechnology applications. We have pushed the cooperativity of a repeat protein to its absolute limit, and our findings provide us with the framework required to exploit this simple and modular architecture to build functional protein-based nanomaterials and to create designer molecular-recognition proteins in synthetic biology and medicine. It will be interesting to explore to what extent other helical repeat proteins are amenable to loop insertion. Given that the current work provides us with a set of guidelines with which we can anticipate the loop energetics, this should be relatively straightforward. We also look forward to exploring the limits of the approach in terms determining what is the maximum length of loop that can be inserted; the sequence composition will be a determining factor for very long loops, as we know that this has a profound effect on both the structure (degree of compactness) and the solubility of intrinsically disordered polypeptides^40^.

## Materials and Methods

### Assembly of tandem-repeat protein genes from single repeat sequences

#### CTPRm25, CTPRm10 and CTPRm10D constructs

All genes were synthesised by GeneArt Invitrogen. Each construct contained BamHI and HindIII restriction sites for subcloning into pRSet for His-tag purification as previously described^29^. The sequences are given in Table S1.

#### CTPRa and CTPRalt constructs

The tandem repeat arrays of CTPRa or CTPR10D (a single CTPR with the ‘10D’ loop sequence) were built by concatemerization of individual CTPRa or CTPR10D using BamHI and BglII sites as previously described^10,29^. Briefly, a single consensus tetratricopeptide repeat (CTPRa1) was purchased as a short double-stranded DNA fragment and inserted into the T7-regulated expression vector pRSET B between the BamHI and HindIII restriction sites (ThermoFisher Scientific). The CTPRa1 fragment was then PCR-amplified using T7 promoter primers. The CTPRa1 PCR product and CTPRa1 pRSET B vector were then digested with BamHI/HindIII and BglII/HindIII restriction enzymes, respectively. The result is two concatamerized CTPRa1 genes froming a CTPRa2 (i.e. two-repeat array). The concatamerization of BamHI and BglII results in an Arg and a Ser after the highly conserved Pro31 of the CTPR sequence. As a result, the CTPRa2 contains the well-studied DPRS loop^30^. This process can be repeated to generate CTPRa proteins of different lengths. Addition of the CTPR10D module generates the CTPRalt constructs. The sequences are given in Table S1.

#### Non-identical CTPR4aX and CTPR4m25X constructs for HDX MS

All constructs were commercially synthesised by IDT as “gBlocks” in the form of a two-repeats array with and without the loop extension. The two-repeat proteins were built up to four-repeat proteins following the concatemerization method used for CTPRa constructs. The sequences of CTPR4aX and CTPR4X are given in Table S5.

### Protein purification

Protein purification of the CTPR proteins was carried as previously described^29^. The 6xHis-tagged constructs were chemically transformed into competent *E. coli* C41 cells by heat shock. Colonies from a selective LB-Amp plate were grown in 2xYT media containing ampicillin (50 μg/mL) at 37 °C, 220 rpm until the optical density (O.D.) at 600 nm reached 0.6. Bacterial cultures were then induced overnight with IPTG (0.5 mM) for 16 h at 20 °C. For large-scale preparations (1 L cultures), cells were centrifugated at 3000 g (4 °C, 10 min) and resuspended in lysis buffer (10 mM sodium phosphate pH 7.4, 150 mM NaCl, 1 tablet of SIGMAFAST protease inhibitor cocktail (EDTA-free), and lysed on an Emulsiflex C5 homogenizer at 15000 psi. The insoluble fraction was separated by centrifugation at 15,000 g at 4 °C for 45 min. Ni-NTA beads were pre-washed once with lysis buffer before incubation with the supernatant from the cell lysate for 1 hr at 4 °C in batch. The loaded beads were washed thrice with phosphate buffer (10 mM sodium phosphate pH 7.4, 150 mM NaCl) containing 30 mM of imidazole to prevent nonspecific interactions. Protein were eluted with phosphate buffer containing 300 mM imidazole and further purified and buffer-exchanged by size-exclusion gel filtration using a HiLoad 16/60 Superdex G75 column (GE Life-Science) in phosphate buffer. Purity was checked by NuPage protein gel (Invitrogen) and proteins were flash-frozen and stored at −80 °C.

For small-scale preparations (15 ml culture), cells were pelleted by centrifugation at 3000 g (4 °C, 10 min) and resuspended in 1 ml of BugBuster Master Mix (Millipore). Ni-NTA were added to the supernatant from the cell lysate for 20 min at 4 °C in batch. The Ni-NTA beads were pre-washed thrice with phosphate buffer (1 mL) containing 30 mM of imidazole. Protein was eluted using phosphate buffer with 300 mM imidazole and dialysed against 50 mM sodium phosphate buffer pH 6.8, 150 mM NaCl. Inclusions bodies were purified from small scale preparations by resuspension of the insoluble pellet in 10 mM sodium phosphate buffer pH 7.4, 150 mM NaCl, 6 M GdmHCl.

Protein concentrations were determined using the calculated extinction coefficients (ExPASy ProtParam)^41^. Molecular weight and purity was confirmed by mass spectrometry (MALDI).

### CD spectroscopy

Circular dichroism experiments were performed as previously described^29^. Briefly, CD measurements were carried on a Chirascan CD spectrometer (Applied Photophysics) in 1 mm pathlength Precision Cells (110-QS, Hellma Analytics) at 25 °C. The CD spectra of all protein samples was measured between 200 nm to 280 nm wavelengths using a 1 nm of bandwidth. Proteins were studied in 50 mM sodium phosphate buffer pH 6.8, 150 mM NaCl at concentrations ranging 5–20 µM. Spectra were acquired at 1 nm intervals every 0.5 s; each reading was taken between three to five times, and the data were averaged.

### Chemical denaturation experiments monitored by fluorescence

Equilibrium denaturation experiments monitored by fluorescence were carried as previously described^42^. In brief, plate measurements were taken on a CLARIOstar Plate Reader (BMG labtech) with a tryptophan detection filter set at 25 °C. Stock solutions of GdmHCl and were dispensed into Corning® 96-well, half area, black polystyrene plates (CLS3993) with a Microlab ML510B dispenser. The protein concentration used was between 0.3 µM and 1 µM. For each protein tested, three replicate sets of serial dilutions were plated consecutively. Final protein concentrations were 0.3–1 µM. Plates were covered with a Microplate Aluminium Sealing Tape and incubated at 25 °C for 1 h shaking.

### Chemical denaturation monitored by CD

Equilibrium denaturation experiments monitored by CD was carried as previously described^29^. The different GdmHCl concentrations were prepared by mixing the apporiate volumes of 50 mM sodium phosphate buffer pH 6.8, 150 mM NaCl, 7 M Gdm HCl and sodium phosphate buffer using a Hamilton Microlab ML510B. The protein concentration used was between 5 µM and 20 µM. Samples were equilibrated at 25 °C for 2 hours. The helical content was followed by changes in ellipticity at 222 nm.

### Analysis of equilibrium denaturation curves

Data were analysed in three different ways: with a two-state model^43^, with a homozipper Ising model or with a heteropolymer Ising model^25^. For two-state analysis, the denaturation curves were fitted directly using Eq. 1.

1 $${{\rm{\lambda }}}_{obs}=\frac{{\alpha }_{N}+{\beta }_{N}[D]+({\alpha }_{D}+{\beta }_{D}[D]).\exp [{m}_{D-N}([D]-{[D]}_{50 \% })]/RT}{1+\exp [{m}_{D-N}([D]-{[D]}_{50 \% })]}$$

where λ_obs_ is the observed signal (fluorescence or CD), α_N_ and α_D_ are the intercepts, and β_N_ and β_D_ are the slopes of the baselines at low (N) and high (D) denaturant concentrations, respectively, [*D*_50%_] is the midpoint of unfolding, [D] is the concentration of denaturant and *m*_D-N_ is a constant that is proportional to the increase in degree of exposure of the protein on denaturation. The free energy of unfolding in water, ${\Delta G}_{D-N}^{{H}_{2}O}$, can then be calculated using Eq. 2:

2 $${\Delta G}_{D-N}^{{H}_{2}O}={m}_{D-N}\cdot {[D]}_{50 \% }$$

To aid comparison of the different datasets, the fluorescence-monitored denaturation curves were normalised by converting each dataset to fraction unfolded (${{\rm{\lambda }}}_{{\rm{U}}}$) using Eq. 3:

3 $${{\rm{\lambda }}}_{{\rm{U}}}=\frac{{{\rm{\lambda }}}_{{\rm{obs}}}-({{\rm{\alpha }}}_{{\rm{N}}}+{{\rm{\beta }}}_{{\rm{N}}}[{\rm{D}}])}{({{\rm{\alpha }}}_{{\rm{D}}}-{{\rm{\alpha }}}_{{\rm{N}}})+({{\rm{\beta }}}_{{\rm{D}}}-{{\rm{\beta }}}_{{\rm{N}}})[{\rm{D}}]}$$

where α_D_/α_N_ are the y-intercept values of the denatured/native baselines and β_D_/β_N_ are the slopes of the denatured /native baselines. Similarly, CD-monitored denaturation curves were individually normalised using Eq. 4:

4 $${{\rm{\lambda }}}_{{\rm{U}}}=\frac{{{\rm{\lambda }}}_{{\rm{obs}}}-{{\rm{\alpha }}}_{{\rm{N}}}}{{{\rm{\alpha }}}_{{\rm{D}}}+({{\rm{\beta }}}_{{\rm{D}}}[{\rm{D}}])-{{\rm{\alpha }}}_{{\rm{N}}}}$$

where α_D_ and α_N_ are the y-intercept values of the denatured/native baselines and β_D_ is the slope of the denatured baseline. This equation allows the slope of the native baselines of the raw data to be preserved in the normalised data.

### Ising model analysis of equilibrium denaturation curves

For the Ising analysis, each fluorescence monitored equilibrium denaturation curve was individually converted to fraction unfolded (${{\rm{\lambda }}}_{{\rm{U}}}$) using Eq. 3. CD-monitored equilibrium denaturation curves were individually normalised using Eq. 4. This equation allows the data to retain the slope of the native baseline and be globally fitted with either homozipper or heteropolymer Ising model. For all constructs, the slopes of their denatured baselines were not significant.

After normalization, the series of curves were globally fitted to either a homozipper or heteropolymer Ising model using the PyFolding package^27^. Both the homozipper and heteropolymer Ising models were constructed as previously described^28,30^. Briefly, each model comprises a one-dimensional linear series of equilibrium constants. These account for the intrinsic folding stability (Δ*G*_i_) and the interfacial energy (Δ*G*_i−1,i_) for each repeated unit in a nearest-neighbour TPR array. The intrinsic stability of the repeating unit has an associated coefficient (*m*) to represent its sensitivity to the external stimulus – in this case chemical denaturant. In the homozipper model all repeated units are identical, whereas in the heteropolymer model, different types of repeat unit can be incorporated.

### Hydrogen/deuterium exchange mass spectrometry

Hydrogen/deuterium exchange mass spectrometry (HDX MS) was performed using a Waters Synapt G2Si equipped with nanoACQUITY UPLC system with H/DX technology and a LEAP autosampler. The final concentrations of proteins in each sample were 5 µM. For each deuteration time, 4 µL complex was equilibrated to 25 °C for 5 min and then mixed with 56 µL D_2_O buffer 50 mM sodium phosphate buffer pH 6.8, 150 mM NaCl, for 0, 0.5, 1, 2, or 5 min. The exchange was quenched with an equal volume of quench solution (3 M guanidinium hydrochloride, 0.1% formic acid, pH 2.66). The quenched sample (50 μL) was injected into the sample loop, followed by digestion on an in-line pepsin column (immobilized pepsin, Pierce, Inc.) at 15 °C. The resulting peptides were captured at 0 °C on a BEH C18 Vanguard pre-column, separated by analytical chromatography (Acquity UPLC BEH C18, 1.0 × 50 mm, Waters Corporation) using a 7–85% gradient acetonitrile in 0.1% formic acid over 7.5 min, and electrosprayed into the Waters SYNAPT G2Si quadrupole time-of-flight mass spectrometer. The mass spectrometer settings and peptide identification methods have been reported previously^44^. The experiments were performed in triplicate, and independent replicates of the triplicate experiment were performed to verify the results. The data were plotted using GraphPad Prism, and fitted to the sum of two exponential to obtain the exchange rates.

## Supplementary information

Supplementary Information

## Data Availability

All data are available from the corresponding author on reasonable request.
