# Supplementary material for: Decoupling a tandem-repeat protein: Impact of multiple loop insertions on a modular scaffold
Source: Sci Rep. 2019 Oct 28;9:15439. doi: 10.1038/s41598-019-49905-4 (PMC6817815; doi:10.1038/s41598-019-49905-4)
Supplement: Supplementary file 1 — Supplementary Information [file 41598_2019_49905_MOESM1_ESM.pdf]

SUPPLEMENTAL INFORMATION

Decoupling a tandem-repeat protein:

Impact of multiple loop insertions on a modular scaffold

Albert Perez-Riba[a,b], Elizabeth Komives[c], Ewan R. G. Main[d],

Laura S. Itzhaki[*]

[a]Department of Pharmacology, University of Cambridge, Tennis Court Road, Cambridge, CB2 1PD, UK

[b]Present address: Donnelly Centre for Cellular & Biomolecular Research, University of Toronto, Toronto, Canada

[c]Department of Chemistry and Biochemistry, University of California, San Diego, 9500 Gilman Drive, La Jolla, CA 92093-0378, USA

[d]School of Biological and Chemical Sciences, Queen Mary University of London, Mile End Road, London, E1 4NS, UK

*Corresponding author: lsi10@cam.ac.uk, telephone +44 1233 334017

Table S1. Amino acid sequences of the CTPRa and multi-loop proteins used in this study. Related to Figure 1.

Protein series	Sequence of the CTPR motifs in each construct
CTPRa	$(AEAWYNLGNAYYKQGDYQKAIEYYQKALELDPRS)_n$
CTPRm25	$(AEAWYNLGNAYYKQGDYQKAIEYYQKALELDPNNSGGGGSGGLVPRGSGSGGGGSGRS)_n$
CTPRm10	$(AEAWYNLGNAYYKQGDYQKAIEYYQKALELDPNNGSLVPRGSRS)_n$
CTPRm10D	$(AEAWYNLGNAYYKQGDYQKAIEYYQKALELDPNNGSDDPRGSRS)_n$
CTPRalt10D	(AEAWYNLGNAYYKQGDYQKAIEYYQKALELDPRS<br>AEAWYNLGNAYYKQGDYQKAIEYYQKALELDPNNGSDDPRGSRS$)_n$

The CTPR proteins used here contain the stabilising Gln-Lys mutation (Cortajarena et al., 2011) instead of the original (Asp-Glu) consensus sequence (Main et al., 2003a). The wild-type inter-loop repeat sequence -PRS- is coloured green. The poly-GS loop sequences of variable length and containing a thrombin cleavage site are coloured blue. The LV to DD mutation in the CTPRm10D series is coloured red.

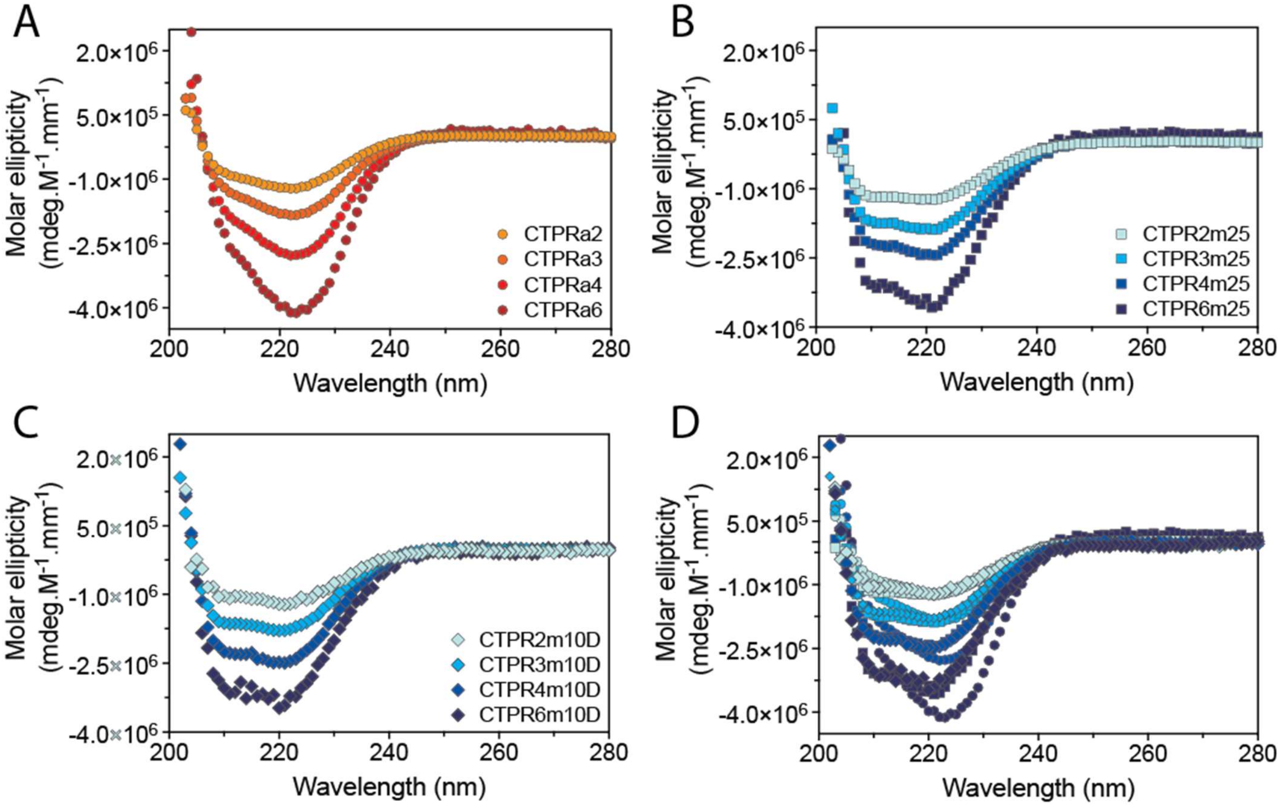

Figure S1: Far-UV CD spectra for: (A) CTPRa series, (B) CTPRm25 series and (C) CTPRm10D series. (D) shows all series plotted on the same graph for comparison. Each trace is the average of three wavelengths scans.

Table S2: Estimated number of TPRs obtained from the ellipticities at 208 nm and 222 nm.

	Estimated number of repeats from 208 nm ellipticity	Estimated number of repeats from 222 nm ellipticity	Average number of repeats from 208 nm & 222 nm ellipticities
CTPR2a	2.0	2.0	2.0
CTPR3a	2.7	3.0	2.9
CTPR4a	3.6	4.6	4.1
CTPR6a	4.6	6.8	5.7
CTPR2m25	2.0	2.0	2.0
CTPR3m25	2.9	3.1	3.0
CTPR4m25	3.7	4.0	3.8
CTPR6m25	5.3	5.8	5.6
CTPR2m10D	2.0	2.0	2.0
CTPR3m10D	3.5	2.9	3.2
CTPR4m10D	4.8	4.1	4.5
CTPR6m10D	6.5	5.3	5.9

The ellipticities at 208 nm and 222 nm were recorded for each protein. Using the ellipticity readings of CTPR2a we determined a value for the ellipticity of a folded repeat. We then divided the ellipticity readings for the other proteins by these values to obtain estimates of the number of folded repeats in each protein.

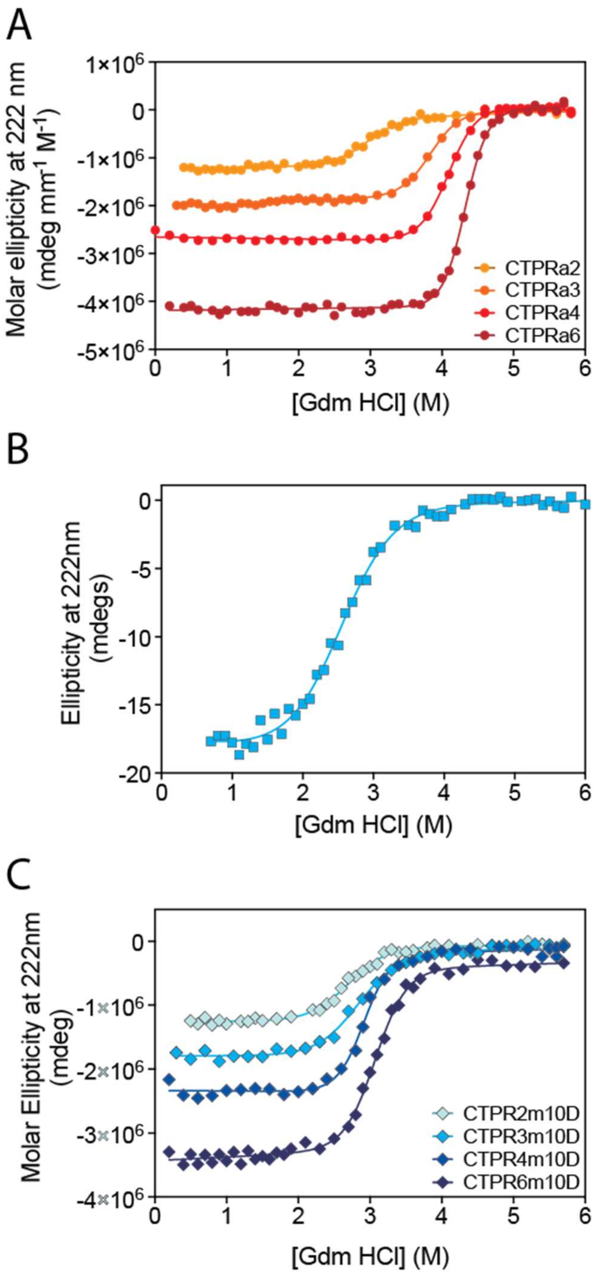

Figure S2: Equilibrium denaturation curves, monitored by ellipticity at 222 nm, for: (A) CTPRa series, (B) CTPR3m25 and (C) CTPRm10D series.

Table S3: Comparison of the two-state fits of the denaturation curves for the CTPRa and CTPRm10D series monitored by fluorescence and CD.

Protein Series	$D_{50\%}$ (M)	*m*-value (kcal mol^{-1} M^{-1})	$\Delta G_{D-N}^{H_2O}$ (kcal mol^{-1})
CTPRa Series – fluorescence monitored (as per Table 1)			
CTPR2a	2.97 ± 0.01	2.1 ± 0.04	6.3 ± 0.1
CTPR3a	3.76 ± 0.01	2.8 ± 0.1	10.4 ± 0.3
CTPR4a	4.04 ± 0.01	3.1 ± 0.1	12.7 ± 0.5
CTPR6a	4.35 ± 0.01	4.0 ± 0. 1	17.3 ± 0.5
CTPRa Series – CD monitored			
CTPR2a	2.96 ± 0.03	2.5 ± 0.3	7.5 ± 0.9
CTPR3a	3.85 ± 0.02	2.9 ± 0.2	11.1 ± 0.7
CTPR4a	4.10 ± 0.01	3.0 ± 0.2	12.2 ± 0.7
CTPR6a	4.32 ± 0.01	3.8 ± 0.2	16.5 ± 0.9
CTPRm10D Series – fluorescence monitored (as per Table 1)			
CTPR2m10D	2.79 ± 0.02	2.2 ± 0.1	5.7 ± 0.3
CTPR3m10D	3.01 ± 0.02	2.3 ± 0.1	6.8 ± 0.3
CTPR4m10D	3.08 ± 0.01	2.4 ± 0.1	7.3 ± 0.2
CTPR6m10D	3.18 ± 0.01	2.8 ± 0.1	8.8 ± 0.3
CTPRm10D Series – CD monitored			
CTPR2m10D	2.70 ± 0.03	2.2 ± 0.2	6.1 ± 0.6
CTPR3m10D	2.81 ± 0.04	2.1 ± 0.2	6.0 ± 0.6
CTPR4m10D	2.91 ± 0.02	3.2 ± 0.4	9.3 ± 1.1
CTPR6m10D	3.10 ± 0.02	2.7 ± 0.2	8.4 ± 0.6

Errors in $D_{50\%}$ and *m*-value are mean standard errors of the fitted variables. Errors in ΔG_{D-N}^{H20} were propagated from these errors.

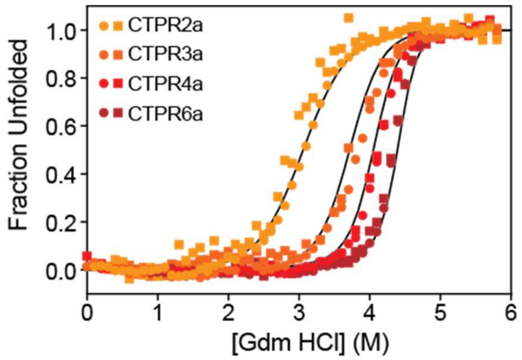

Figure S3: Equilibrium denaturation curves monitored by fluorescence and CD for the CTPRa series fitted to a Homozipper Ising Model. Each denaturation was converted to fraction unfolded (Fluorescence - filled circles, CD - filled squares).

Table S4: Parameters obtained from fitting the CTPRa series of protein to a Homozipper Ising model.

ΔG_{i} (kcal mol^{-1})	-1.1 ± 0.04
m_{i} (kcal mol^{-1} M^{-1})	1.0 ± 0.02
ΔG_{ij} (kcal mol^{-1})	-3.7 ± 0.07

Errors reported are the standard errors of the fitted variables.

Table S5: Amino acid sequences of the mutant CTPR4a and CTPR4m25 proteins (CTPRa4X and CTPR4m25X, respectively) used in HDXMS experiments (associated with Figure 3).

**Protein**	**Sequence**
CTPR4aX	MRGSHHHHHHGLVPRGS<br>AEAWYNLGNAYYKQGDYQKAIEYYQKALELDPRS<br>AEAWYNLGQAYYKQGDYQRAIEYYNRALELDPRS<br>AEAWFNLGNAFYKQGDYQKAIDYYQNALELDPRS<br>AEAWFNLGNAYYQQGDYQKAIEYYNKALELDPRS
CTPR4m25X	MRGSHHHHHHGLVPRGS<br>AEAWYNLGNAYYKQGDYQKAIEYYQKALELDPNNGSGGGGSGGLVPRGSGSGGGGSGRS<br>AEAWYNLGQAYYKQGDYQRAIEYYNRALELDPNNGSGGGGSGGLVPRGSGSGGGGSGRS<br>AEAWFNLGNAFYKQGDYQKAIDYYQNALELDPNNGSGGGGSGGLVPRGSGSGGGGSGRS<br>AEAWFNLGNAYYQQGDYQKAIEYYNKALELDPNN

The CTPR proteins used here contain the stabilising Gln-Lys mutation (Cortajarena et al., 2011) instead of the original (Asp-Glu) consensus sequence (Main et al., 2003a). The wild-type inter-loop repeat -PRS- is coloured green. The poly-GS loop sequences of variable length and containing a thrombin cleavage site are coloured blue. The mutations to the standard CTPR sequences in the CTPR4ml25 protein are coloured red.

Table S6. Details of the four peptides used to report the hydrogen exchange at each one of the four repeats of CTPR4a and CTPR4ml25.

Peptide	Corresponding symbol in Figure 3	Position in CTPRa4 sequence (residue number)	Amino acid sequence
Peptide 1	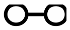	28- 40	YYKQGDYQKAIEY
Peptide 2	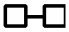	62- 73	YYKQGDYQRAIE
Peptide 3	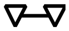	96- 108	FYKQGDYQKAIDY
Peptide 4	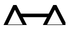	131- 141	YQQGDYQKAIE

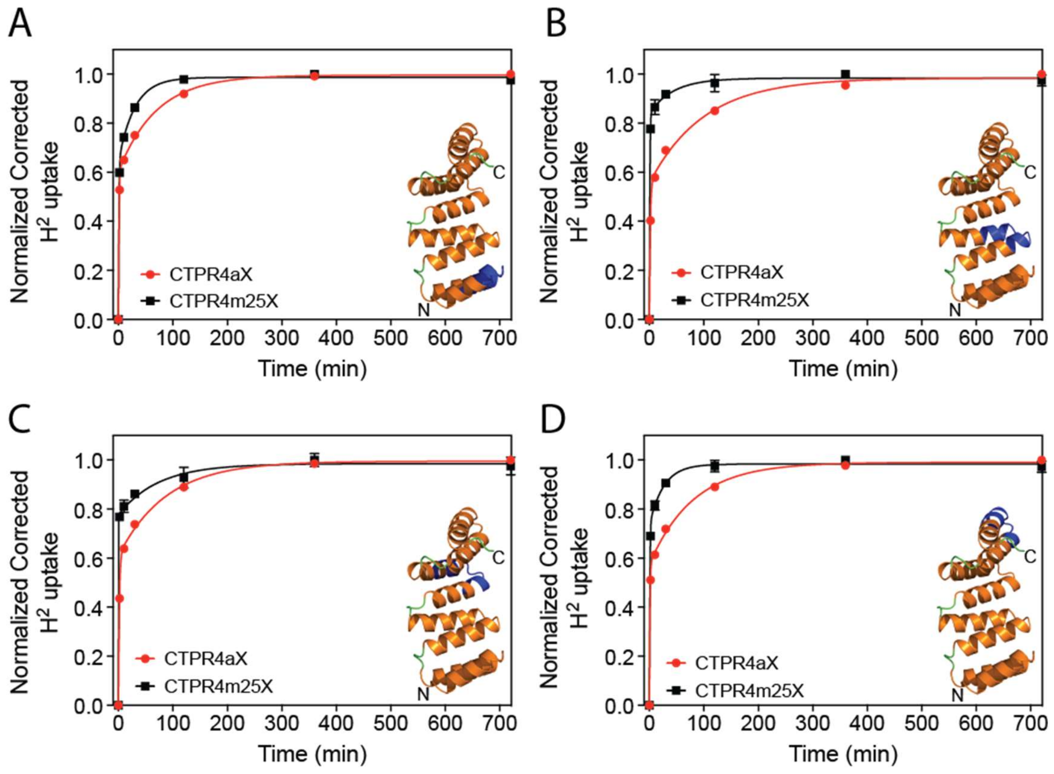

Figure S4: Normalised deuterium uptake as a function of time for CTPR4aX and CTPR4m25X proteins. Each plot shows the reporter peptide from each of the four repeats in the proteins (described in Figure 3, Table S6 and shown in the inset structural model in blue): (A) repeat 1, (B) repeat 2, (C) repeat 3, (D) repeat 4. Each dataset is fitted to the sum of two exponential phases. The deuterium uptake was corrected for back-exchange and normalised as described in Materials and Methods.

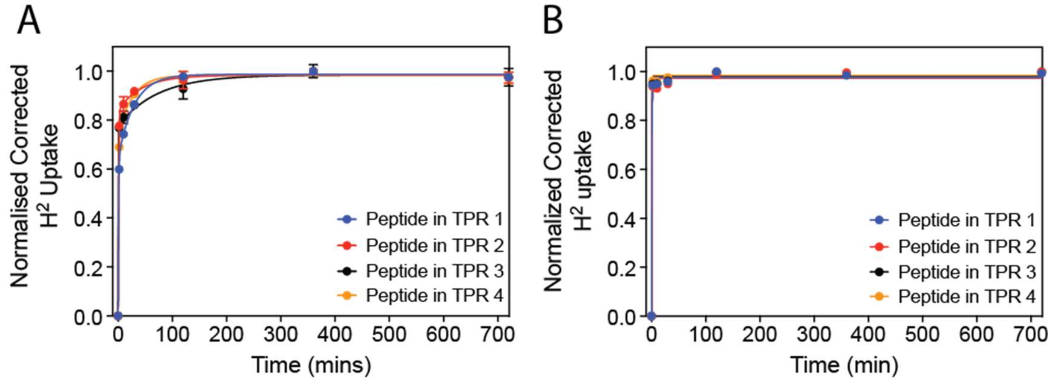

Figure S5: Normalised deuterium uptake as a function of time for CTPR4m25X in native buffer (A) and 5 M Urea (B). Each plot shows all reporter peptides from each TPR motif (described Figure 3). Each dataset is fitted to the sum of two exponential phases. The deuterium uptake was corrected for back-exchange and normalised as described in Materials and Methods.

Table S7: Equilibrium denaturation curves monitored by Fluorescence and CD for the CTPRalt loop proteins and the CTPR single loop proteins.

Protein Series	$D_{50\%}$ (M)	*m*-value (kcal mol^{-1} M^{-1})	$\Delta G_{D-N}^{H_2O}$ (kcal mol^{-1})
CTPR alternating loop proteins – Fluorescence monitored			
CTPR2m10D	2.79 ± 0.02	2.2 ± 0.1	5.7 ± 0.3
CTPR4alt10D	3.59 ± 0.01	2.44 ± 0.05	8.8 ± 0.2
CTPR6alt10D	3.76 ± 0.01	2.62 ± 0.05	9.9 ± 0.2
CTPR alternating loop proteins – CD monitored			
CTPR2m10D	2.70 ± 0.03	2.2 ± 0.2	6.1 ± 0.6
CTPR4alt10D	3.52 ± 0.04	2.3 ±0.3	8.2 ± 0.9
CTPR6alt10D	3.72 ± 0.01	2.7 ± 0.1	10.0 ± 0.5
CTPR single loop proteins – Fluorescence monitored			
CTPR6L1-2	4.30 ± 0.01	3.36 ± 0.07	14.4 ± 0.3
CTPR6L3-4	4.01 ± 0.01	3.27 ± 0.05	13.1 ± 0.2
CTPR single loop proteins – CD monitored			
CTPR6L1-2	4.25 ± 0.02	3.4 ± 0.3	14.4 ± 1.1
CTPR6L3-4	3.97 ± 0.02	3.9 ± 0.3	15.6 ± 1.4

The data are fitted to a two-state model. Errors in $D_{50\%}$ and *m*-value are mean standard errors of the fitted variables. Errors in ΔG_{D-N}^{H20} were propagated from the errors obtained from the mean standard errors of the fitted variables.

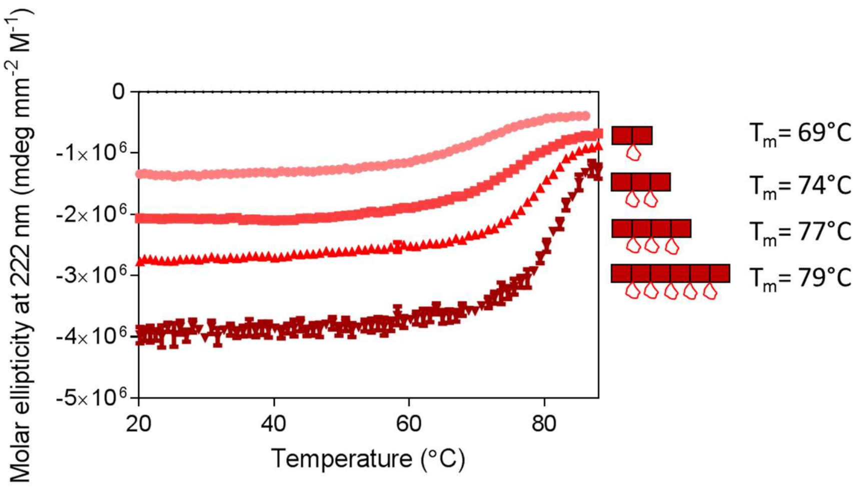

Figure S6: Thermal denaturation curves for the CTPRm10D series monitored by CD.
